# Refractory Lung Diseases: From Cellular Structures, Molecular Mechanisms to Therapeutic Strategies

**DOI:** 10.1002/pdi3.70040

**Published:** 2026-03-19

**Authors:** Yiheng Qiu, Xiangyu Dong, Yi Zhu, Nicole Miranda, Awele Chukwura, Yan Peng, Sarina Zhao, Michelle Xiang, Peyton McGuire, Ava Zou, Lucas Zhang, Jiaming Fan, Linghuan Zhang

**Affiliations:** ^1^ Ministry of Education Key Laboratory of Diagnostic Medicine, and Department of Clinical Biochemistry School of Clinical Laboratory Medicine Chongqing Medical University Chongqing China; ^2^ Department of Orthopaedic Surgery and Rehabilitation Medicine Molecular Oncology Laboratory The University of Chicago Medical Center Chicago Illinois USA; ^3^ Pritzker School of Medicine The University of Chicago Medical Center Chicago Illinois USA; ^4^ Department of Cardiology the Affiliated University‐town Hospital of Chongqing Medical University Chongqing China; ^5^ Stem Cell Biology and Therapy Laboratory of the Pediatric Research Institute the National Clinical Research Center for Child Health and Disorders, and Ministry of Education Key Laboratory of Child Development and Disorders National Clinical Research Center for Children and Adolescents' Health and Diseases, and Ministry of Education Key Laboratory of Child Development and Disorders Children's Hospital of Chongqing Medical University Chongqing China

**Keywords:** alveolar microenvironment homeostasis, antifibrotic therapy, antioxidant therapy, refractory lung diseases (RLDs)

## Abstract

Refractory lung diseases (RLDs) encompass a spectrum of progressive pulmonary disorders, including acute respiratory distress syndrome (ARDS), bronchopulmonary dysplasia (BPD), and idiopathic pulmonary fibrosis (IPF). These conditions are defined by poor responsiveness to current therapeutic interventions and pose substantial clinical challenges, primarily due to their high morbidity and mortality rates. This review synthesizes the current understanding of the molecular mechanisms underlying RLDs and explores promising therapeutic strategies. A core feature of RLD pathogenesis is the disruption of alveolar microenvironmental homeostasis, which triggers a bidirectional vicious cycle between structural damage and disease progression. This homeostatic collapse is driven by interconnected pathological networks—including oxidative stress coupled with mitochondrial dysfunction, inflammatory immune dysregulation, and mechanical stress‐induced extracellular matrix (ECM) remodeling—all of which are elaborated in this review. Collectively, these pathological processes contribute to therapeutic resistance. Based on these mechanistic insights, potential therapeutic approaches are discussed, such as antioxidant therapies (e.g., the mitochondria‐targeted antioxidant Mitoquinone mesylate [MitoQ]) and antifibrotic agents (e.g., pirfenidone, nintedanib, as well as emerging strategies targeting Wnt pathway modulation). Additionally, the critical significance of early diagnosis and personalized precision medicine is emphasized. Future research should focus on a deeper characterization of the dynamic alterations within the alveolar microenvironment under pathological conditions, with the aim of developing more precise diagnostic tools and targeted therapeutic strategies. Ultimately, the therapeutic goal for RLDs should shift from mere symptom management to achieving pathological reversal.

## 1 Introduction

Chronic respiratory diseases (CRDs) represent a leading global cause of morbidity and mortality. The latest data from the Global Burden of Disease Study 2019 (GBD 2019) indicate that CRDs constitute the third leading cause of death worldwide and are responsible for 4.0 million (95% uncertainty interval 3.6–4.3 million) deaths, posing a severe threat to human health and life expectancy [1 ]. Although most pulmonary disorders can be effectively managed with current therapeutic approaches, refractory lung diseases (RLDs) exhibit poor responsiveness to existing pharmacological regimens and comprehensive supportive care, presenting as a diverse group of pulmonary lesions characterized by progressive deterioration. Consequently, RLDs pose significant clinical challenges. These diseases are marked by unfavorable prognoses and a substantially diminished quality of life, primarily manifesting as progressive destruction of alveolar architecture and therapeutic resistance. Representative conditions include acute respiratory distress syndrome (ARDS), bronchopulmonary dysplasia (BPD), idiopathic pulmonary fibrosis (IPF), bronchiolitis obliterans (BO), and difficult‐to‐treat asthma (DA) [2]. Despite recent incremental progress in interventions such as targeted therapies and cell‐based treatments, significant limitations persist, including insufficient specificity and inadequate therapeutic efficacy. Therefore, identifying novel molecular mechanisms and therapeutic strategies is paramount.

The clinical management of RLDs faces a dual challenge: the spatial heterogeneity of the alveolar microenvironment compromises early detection [3], and single‐target therapies fail to modulate multi‐pathway networks, as seen in ARDS where corticosteroids cannot reverse barrier damage or microthrombosis [4, 5]. These limitations highlight the need for integrated strategies targeting core pathological networks (e.g., oxidative stress and immune dysregulation), which are elaborated in the subsequent section on pathological mechanisms (Section 4) [6, 7, 8]. This review aims to synthesize these insights and explore clinical interventions.

## 2 Alveolar Structure and Function: The Cornerstone of Respiratory Physiology

Alveoli function as the core functional units for gas exchange in mammalian lungs. They are numerous small sac‐like structures formed by the terminal dilatations of bronchioles. Their intricate three‐dimensional architecture and dynamically balanced regulatory networks constitute the cornerstone of vital respiratory activity. Recent research has increasingly revealed potential links between pathological alterations in RLDs and the inherent heterogeneity of alveolar structures. This section systematically delineates the biological foundations of the alveolus from two dimensions—cellular composition with its ultrastructure and functional characteristics—to establish a framework for understanding its potential association with RLDs.

### 2.1 Alveolar Cellular Composition and Ultrastructure

**FIGURE 1:**
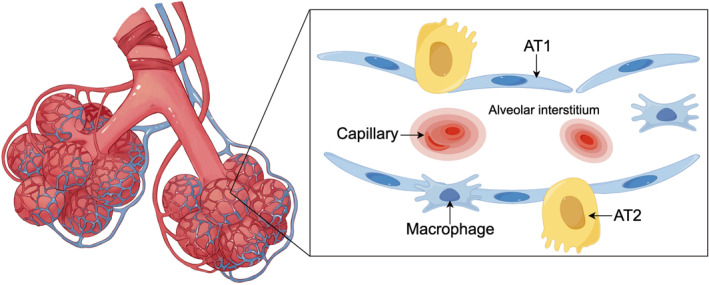
Cellular components that are involved in the pathogenic development of refractory lung diseases (RLDs). Alveolar clusters gather at the terminal ends of the bronchioles, their surfaces enveloped with abundant capillaries, and microscopically, alveolar type I (AT1) and II (AT2) alveolar epithelial cells line the alveolar spaces, with AT1 cells contributing to gas exchange and AT2 cells involved in surfactant production and repair. Macrophages patrol the alveolar environment, playing roles in immune surveillance and inflammation modulation. Capillaries closely associate with the alveolar epithelium, facilitating gas exchange but also serving as sites for inflammatory and fibrotic responses. The alveolar interstitium, the connective tissue matrix between cells, provides structural support and can undergo remodeling during disease progression, influencing the mechanical properties of the lung. Together, these cellular components interact in complex manners to drive the development and progression of RLDs.

An alveolus represents a complex microenvironment formed by heterogeneous cell populations and the extracellular matrix (ECM). Based on function, its cellular organization can be broadly stratified into three layers: the alveolar lumen interface layer, the basement membrane (BM) and interstitial layer, and the capillary endothelial layer. Cells within these layers interact extensively to collectively maintain alveolar surface activity and structural integrity (Figure 1). The principal cellular constituents include:

### 2.2 Type I Alveolar Epithelial Cells

Alveolar Type I epithelial (AT1) cells are flat, squamous cells, constituting only approximately 10% of the total lung cell population. AT1 cells are the primary functional units for gas exchange and they cover roughly 95% of the alveolar surface area with their highly branched, flattened cytoplasm (ranging from 50 to 100 μm in diameter; individual cell coverage up to 5098 μm^2^). They form intimate connections with surrounding capillary plexuses, establishing a continuous blood–gas barrier (BGB) [9, 10]. Immunostaining with AT1‐specific antibodies reveals high expression of integral membrane proteins Caveolin‐1 and the water channel AQP‐5 on their cytoplasmic side, indicating that AT1 cells confer high permeability to the lung and regulate alveolar water homeostasis [11]. Traditionally viewed as terminally differentiated and devoid of proliferative capacity, AT1 cell regeneration was thought to depend entirely on differentiation from alveolar type II epithelial (AT2) cells [12]. However, recent investigations suggest that mature AT1 cells may retain cellular plasticity. In alveolar regeneration induced by pneumonectomy, they can proliferate and give rise to AT2 cells [13].

### 2.3 Alveolar Type II Epithelial Cells

AT2 cells, also known as cuboidal secretory cells (volume 450–900 μm^3^), comprise approximately 60% of the total alveolar epithelial cell number but cover only 5% of the surface area. They are responsible for synthesizing, secreting, and recycling surfactant components and mediating the repair of the damaged alveolar epithelium [14]. Each AT2 cell anchors to the surfactant layer via microvilli (height 1.5 μm) and expresses β‐adrenergic receptors on its surface. Binding of β‐agonists to these receptors stimulates the translocation of lamellar bodies (LBs), leading to the rhythmic exocytotic release of dipalmitoylphosphatidylcholine (DPPC) and hydrophobic surfactant proteins (SP‐B, SP‐C) into the alveolar lumen [15, 16, 17]. This surfactant layer is critically important for lowering alveolar surface tension, preventing end‐expiratory alveolar collapse, maintaining normal lung function in neonates, controlling inflammation, and defending against pulmonary infections. Moreover, AT2 cells serve as the alveolar stem cell reservoir. Driven by the Wnt/β‐catenin pathway, AT2 cells undergo depolarization and cytoskeletal remodeling to differentiate into AT1 cells during injury repair [18], playing a pivotal role in restoring alveolar epithelial integrity.

### 2.4 Basement Membrane

The basement membrane (BM) is a thin, acellular structure composed of a highly specialized extracellular matrix (sECM), situated at the basal aspect of cells. It serves both to separate cells from the interstitial stroma and to mediate their functional connection [19]. The BM is a bilayered reticular structure (total thickness 80–100 nm), primarily composed of type IV collagen (constituting about 60% of its dry weight), laminins (predominantly laminin‐511 and laminin‐111), and nidogen‐1. Within the barrier region, the epithelial BM and endothelial BM fuse tightly, forming a trilaminar “sandwich” structure (epithelial BM‐common layer‐endothelial BM). This fusion ensures an ultrathin air–blood distance not exceeding 0.2–0.4 μm^11^. Concurrently, alveolar stability relies on surfactant membranes reducing surface tension at the air–liquid interface. The BM facilitates epithelial‐mesenchymal mechanotransduction, regulating cell chemotaxis and polarity through integrins such as α3β1 and GTPases [20, 21, 22], thereby stabilizing alveolar architecture.

### 2.5 Capillary Endothelial Cells

Alveolar capillaries are lined by continuous endothelium. Endothelial cells project thin lamellae from a central cell body containing the nucleus, forming a dense, continuous hexagonal capillary network (segment length 6–8 μm), with a single endothelial cell covering approximately 1350 μm^2^ of the surface area [10]. The secretory function of vascular endothelial cells is linked to coagulation control. Distinct from systemic arterial and venous endothelium, alveolar capillary endothelial cells lack Weibel–Palade bodies (rod‐shaped secretory storage organelles containing von Willebrand factor protein) [23, 24]. The absence of Weibel–Palade body secretory capacity reflects their specialized antithrombotic phenotype. Endothelial cells are anchored to each other via tight junctions (*zonula occludens*, ZO) and express the transmembrane water channel *AQP1* [25]. *AQP1* facilitates transepithelial water transport in the lung, contributing to airway hydration, defense, and the absorption of excess alveolar fluid. The potential clinical application of this water‐clearance mechanism for treating pulmonary edema warrants further investigation.

### 2.6 Interstitial Cell Populations

The key interstitial cell populations within the alveolus include fibroblasts, pericytes, and alveolar macrophages, playing indispensable roles in maintaining tissue integrity, providing structural support, and orchestrating immune functions. Fibroblasts, primarily secreting elastin and collagen, are implicated in pulmonary fibrosis through their regulation of matrix remodeling via the TGF‐β/Smad pathway [26]. Pericytes are heterogeneous mesenchymal cells closely opposed to the abluminal surface of pulmonary capillaries, providing structural and biochemical support. Emerging research suggests pericytes may play significant roles in some RLDs [27]. Alveolar macrophages are the largest free‐floating cells (volume 2491 μm^3^), extending surface pseudopods over the surfactant membrane. They recognize signaling molecules on apoptotic cell surfaces, such as CX3CL1 (fractalkine), nucleotides (ATP and UTP), sphingosine‐1‐phosphate (S1P), and lysophosphatidylcholine (LysoPC) [28], executing efferocytosis to clear apoptotic bodies.

## 3 Safeguarding Alveolar Structure and Function: The Blood–Gas Barrier

The alveolus constitutes the minimal functional unit for gas exchange in the lung. During the exchange of gases between the internal and external environments, gases inevitably traverse an interfacial barrier separating the liquid plasma phase containing red blood cells within the capillary from the gaseous phase within the alveolus that communicates with the external environment. That sandwiched between these two phases is the cellular/tissue phase [29]. Three distinct forces act to maintain the spherical structure of the alveolus: circumferential wall tension induced by transmural pressure, surface tension at the alveolar lining layer, and tension within the alveolar wall tissue components [30]. The passage of gases readily disturbs the forces at these phase interfaces, potentially causing structural changes in the intermediate (cellular/tissue) phase. However, in normal alveolar tissue, gas exchange does not typically result in alveolar collapse or rupture. Therefore, a stabilizing “barrier” must exist at the phase interface to counteract perturbations generated during gas transit. This critical barrier is termed as the blood–gas barrier (BGB).

The BGB is composed of the alveolar surface liquid layer (secreted by AT2 cells), the plasma membrane of AT1 cells, the plasma membrane of endothelial cells, and the fused ECM formed by the two basement membranes. It serves as the structural interface for gas exchange between oxygen in the alveolar airspace and carbon dioxide in the blood within capillaries. Characterized by an immense surface area (50–100 m^2^) and an extremely thin total thickness (∼0.2–0.5 μm), this large yet delicate structure possesses inherent fragility. Fortunately, type IV collagen, a major constituent of the basement membrane, plays a crucial role in maintaining membrane toughness to prevent mechanical failure.

Remarkably, even when simultaneous rupture of the capillary endothelium and alveolar epithelium occurs, the ECM can remain intact at the ultrastructural level [31], indicating that the ECM is likely the key element responsible for the strength of the BGB. Within the BM, the “chicken‐wire” distribution of type IV collagen not only provides immense tensile strength but also effectively resists the circumferential wall tension generated by elevated capillary transmural pressure [32]. Consequently, alterations in the content or distribution of type IV collagen in the alveolar basement layer predispose the organism to alveolar collapse and rupture. Similarly, increased type IV collagen content or BM thickening impairs gas exchange and airway hydration, commonly observed in clinical conditions such as interstitial pulmonary edema or pulmonary fibrosis.

However, the mechanical resistance provided by type IV collagen alone is insufficient. During the end‐expiratory phase, as intra‐alveolar gas pressure decreases, the pressure gradient causes capillaries to bulge into the alveolar space. To counteract this, AT2 cells secrete surfactant to reduce surface tension at the air–liquid interface, thereby preserving alveolar structural integrity at end‐expiration [30]. Furthermore, surfactant proteins SP‐B and SP‐C significantly enhance the surface pressure and mechanical stability of the surfactant monolayer by specifically binding phospholipids and inducing the formation of the liquid‐expanded (Le) phase, thereby disrupting the tight packing of the liquid‐condensed (Lc) phase, which effectively prevents alveolar collapse [33].

## 4 RLDs and the Alveoli: A Pathological Interplay of Mutual Reinforcement

The integrity of the alveolar structure is fundamental for the execution of normal respiratory functions, whereas the architectural design of the alveolus itself is inherently adapted to support its unique biological activities. This bidirectional relationship between the structure and function is crucial for sustaining fundamental life processes. It reveals that pathological alterations in specific tissue structures are not merely consequences of disease but can also act as initiating or exacerbating factors. In the context of RLDs, the relationship between alveolar pathology and disease progression is not a simple linear cause‐and‐effect; instead, it forms an “initiation‐amplification” bidirectional interaction network. This network encompasses both the “ignition effect” of specific alveolar lesions on disease onset and the “remodeling retaliation” exerted by disease progression on the alveolar microenvironment. From this perspective, dissecting the distinctions and commonalities between them is key to overcoming therapeutic resistance. This section aims to investigate the pathological alterations within the alveolus during RLDs and further integrate the core pathological mechanisms.

### 4.1 Pathological Alterations in the Alveolus During RLDs

Initial alveolar injury serves as a common initiating factor for various respiratory diseases, with mechanisms exhibiting specificity and targeting. The type of initial injury and its underlying molecular mechanisms dictate the subsequent disease‐specific phenotype. As elucidated in the description of fundamental alveolar functions, alveolar collapse or rupture often results from mechanical damage. For example, degradation of ZO proteins within the actin cytoskeleton compromises the BGB, triggering alveolar edema and neutrophil infiltration in patients with ARDS, thereby forming the pathological hallmark of ARDS‐pulmonary interstitial edema [34]. Beyond BGB damage, BM rupture is also a significant factor in the development of RLDs. During the repair of ruptured BM, senescence of AT2 cells leads to fibroblast activation and excessive collagen deposition, ultimately culminating in IPF.

Abnormal secretion of airway cellular products constitutes another element of pathological alveolar changes. BPD is the most common lung disease in preterm infants, with its incidence inversely correlated with gestational age and birth weight [35]. This disease arises due to the incomplete differentiation of fetal AT2 cells and LBs during late gestation, resulting in surfactant deficiency. This deficit leads to reduced alveolarization, initiating BPD. In contrast to the secretory defect characteristic of BPD, excessive airway mucus secretion forms mucus plugs that are strongly associated with indicators of airflow obstruction in asthma [36]. Chronic manifestations may contribute to difficult‐to‐treat asthma (DA).

As evident from the above, RLDs triggered by alveolar pathologies exhibit initiating specificity (involving anomalies in single cell types or molecular pathways) and spatiotemporal limitation (early lesions can be localized). Early alveolar supportive therapy can potentially redirect the course of the disease. Therefore, developing efficient and rapid screening methods for such lesions, enabling preemptive intervention to suppress corresponding alveolar pathologies and exclude confounding etiological factors, represents a critical direction for managing RLDs stemming from alveolar damage.

### 4.2 Pathological Mechanisms Linking RLDs to the Alveolus and Their Clinical Translation

**FIGURE 2:**
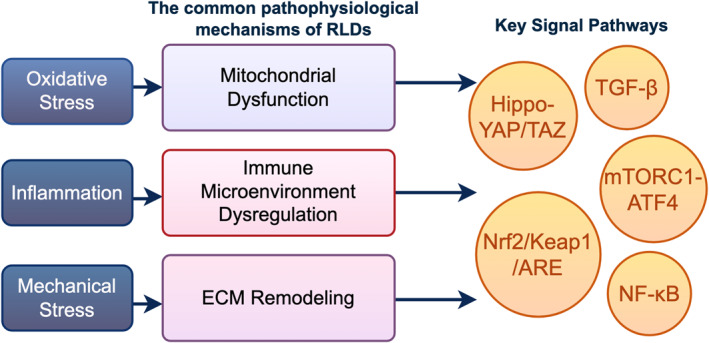
Essential signaling pathways that are implicated in the development of respiratory lung diseases (RLDs). The development of RLDs primarily involves three critical pathological processes: mitochondrial dysfunction, induced by oxidative stress; immune microenvironment dysregulation, caused by inflammation; and extracellular matrix (ECM) remodeling, driven by mechanical stress. These pathological processes further activate key signaling pathways such as Hippo‐YAP/TAZ, mTORC1‐ATF4, Nrf2/Keap1/ARE, and NF‐κB, which collectively participate in the initiation and progression of RLDs. This provides a theoretical basis for elucidating the pathogenesis of RLDs and designing targeted therapeutic strategies.

The disruption of alveolar homeostasis serves as a common pathological nexus for multiple RLDs. During RLD progression, systemic pathological alterations inflict retaliatory effects on the alveolar structure, characterized by network‐like interactions (involving multicellular crosstalk and pathway intersections) and irreversibility. Within this intricate network, identifying shared and divergent alveolar‐related mechanisms across RLDs may constitute the core strategy for guiding personalized treatment, directing precise clinical pharmacotherapy, and improving therapeutic resistance (Figure 2).

### 4.3 Shared Molecular Mechanisms: A Cross‐Disease Pathological Core Network

As introduced earlier, the pathogenesis of RLDs revolves around the collapse of alveolar homeostasis [3, 15]. The core pathological networks, namely oxidative stress, inflammatory‐immune dysregulation, and mechanical stress remodeling, are interconnected and drive disease progression across different RLDs, as detailed in the following subsections. These pathways are visualized in Figure 2, illustrating their interplay.

#### 4.3.1 Oxidative Stress and Mitochondrial Dysfunction

Aberrant accumulation of mitochondrial reactive oxygen species (mtROS) drives the pathological progression of RLDs. In IPF, mtROS accelerates AT2 cell senescence via p53 activation, playing a pivotal role in the development of pulmonary fibrosis [6, 7]. In ARDS patients with infection, neutrophils elevate mtROS levels, regulating the excessive release of neutrophil extracellular traps (NETs) during lung injury, accelerating the release of myeloperoxidase (MPO) and superoxide dismutase (SOD). These NET‐associated proteins exhibit high cytotoxicity, inducing endothelial and epithelial cell death and damaging host proteins and the cellular matrix, directly disrupting the blood–gas barrier [37, 38]. For preterm infants, hyperoxia‐induced oxidative stress triggers a burst of reactive oxygen species (ROS), leading to endothelial and epithelial destruction and loss of alveolar‐capillary barrier integrity, thereby increasing the risk of BPD [39, 40, 41]. Excessive airway mucus secretion is a significant factor in the morbidity and mortality of DA patients [42]. Inhalation of air containing allergenic particles generates substantial ROS, stimulating increased mucus secretion. Activation of the Nrf2/Keap1/ARE signaling pathway exerts anti‐inflammatory and antioxidant effects, reducing airway eosinophils and mucus hypersecretion [42, 43]. Downregulation of this pathway diminishes the body's anti‐inflammatory and antioxidant capacity, exacerbating airway epithelial injury [44, 45].

These oxidative stress mechanisms underscore therapeutic targets, as discussed in Section 5.1 [46, 47]. Collectively, these findings indicate that the pathogenic mechanisms of many RLDs are closely associated with the suppression of the Nrf2/Keap1/ARE signaling pathway and downregulation of antioxidant enzymes (e.g., SOD2). This suggests that targeting the Nrf2/Keap1/ARE pathway may represent a potential strategy for addressing therapeutic resistance in RLDs. Reducing airway ROS levels emerges as a core therapeutic target for RLDs. Therefore, mitochondria‐targeted antioxidants (e.g., Mitoquinone mesylate [MitoQ]) [46] or agonists targeting the Nrf2/Keap1/ARE pathway could serve as cross‐disease therapeutic strategies.

#### 4.3.2 Dysregulation of the Inflammatory‐Immune Microenvironment

Inflammatory cascades mediated by pro‐inflammatory cytokines such as TNF‐α, IL‐1β, IL‐6, and TGF‐β are central mediators driving fibrosis. In IPF patients, M1 macrophages release increased IL‐6, stimulating macrophage polarization toward a profibrotic M2 phenotype. IL‐6 also acts as an autocrine growth factor, promoting fibroblast secretion of IL‐6 in a proliferative loop. Patient IL‐6 levels correlate with disease severity, leading to the irreversible deterioration of pulmonary fibrosis [8, 48]. The inflammatory cascade in ARDS involves multiple signaling pathways, including NF‐κB, mitogen‐activated protein kinase (MAPK), and JAK/STAT [49]. Using the NF‐κB pathway as an example, tumor necrosis factor receptor (TNFR) or surface receptor and IL‐1 receptor (IL‐1R) activation stimulates the NF‐κB pathway. Phosphorylation of the NF‐κB inhibitory protein (IκB) bound to NF‐κB releases NF‐κB, ultimately recruiting inflammatory cells and releasing cytokines such as IL‐1 and IL‐6. This cascade amplifies the NF‐κB inflammatory response pathway, causing persistent lung tissue injury [50]. The terminal pathway in BO involves lung tissue fibrosis leading to airway narrowing and obstruction, implicating excessive fibroblast proliferation. Research indicates that the activation of the alveolar macrophage pyroptosis pathway upon lung injury releases IL‐18 and IL‐1β, stimulating pulmonary fibrosis and gasdermin D protein‐mediated alveolar‐capillary barrier disruption [51]. In IPF patients, oxidative stress leads to massive ROS release, activating transforming growth factor‐β1 (TGF‐β1)‐mediated epithelial‐mesenchymal transition (EMT). This process significantly impacts airway remodeling in asthma, contributing to the progressive decline in lung function observed in IPF patients [52].

It is evident that inflammatory cytokines themselves are not the primary initiators of inflammatory cascades, but their presence invariably accompanies the cyclic amplification of inflammatory responses. This relentless amplification cycle ultimately leads to irreversible alveolar tissue damage (e.g., irreversible destruction of the BGB, alveolar tissue fibrosis). Therefore, whether interrupting the cycle mediated by inflammatory cytokines (e.g., IL‐6, TGF‐β) before irreversible RLD lesions occur can reverse the inflammatory response and overcome “therapeutic resistance” stemming from imprecise targeting, which warrants further investigation.

#### 4.3.3 Mechanical Stress and Matrix Mechanotransduction Remodeling

Cells perceive physical microenvironmental changes, such as ECM stiffness or adhesiveness, through the mechanical stress signaling hub Hippo‐YAP/TAZ. This activation subsequently triggers downstream pathways regulating cell morphology and inducing mesenchymal stem cell differentiation, revealing the critical regulatory role of mechanical stress in shaping tissue morphology and homeostasis [53]. Increased nuclear expression of YAP and TAZ, mediators of the Hippo pathway, correlates with enhanced AT2 activity. Conversely, YAP/TAZ deficiency in AT2 cells impairs alveolar epithelial regeneration and leads to prolonged fibrotic lesions during bacterial pneumonia [54], suggesting that this pathway participates in the resolution of pulmonary inflammation, regeneration of alveolar epithelial cells, and reconstruction of the BGB.

In alveolar epithelial cells and lung fibroblasts of IPF patients, nuclear localization of YAP/TAZ is significantly increased, which is closely associated with downregulated expression of the Hippo pathway kinases MST1/2 [56]. Furthermore, mechanical stress activates pathways such as RhoA/ROCK, leading to the inhibition of LATS (large tumor suppressor kinase) activity, dephosphorylation and nuclear translocation of YAP/TAZ, and subsequent binding to TEAD (TEA domain transcription factor) to induce profibrotic gene expression (e.g., *CTGF*, *CYR61*). YAP1 also promotes fibroblast‐to‐myofibroblast transformation (characterized by α‐SMA positivity) via Twist1 activation, and synergizes with TGF‐β signaling to enhance Smad2/3 phosphorylation and drive excessive collagen and fibronectin deposition. Additionally, YAP/TAZ interacts with the PI3K/AKT/mTOR pathway, regulating abnormal alveolar epithelial cell proliferation, migration, EMT, and tissue remodeling [55]. In murine AT2 cells, mutation or upregulated expression of YAP/TAZ significantly influences NF‐κB expression levels by targeting IκBα, indicating this pathway's relevance to the inflammatory cascade in ARDS [54].

BPD is frequently complicated by pulmonary hypertension (PH). As the most common and severe respiratory diseases in preterm infants, the mechanisms underlying their co‐occurrence remain elusive [56]. However, a growing body of research suggests the Hippo signaling pathway is critically involved in early lung development and may also mediate PH pathogenesis [57, 58, 59, 60]. Although research on this pathway in BPD is still limited, recent studies have confirmed the essential role of the Hippo‐YAP/TAZ signaling axis in lung development [56]. This signaling pathway holds significant potential as a crucial target for elucidating BPD‐related pathological mechanisms and developing novel therapeutic approaches.

Beyond its interplay with the Hippo pathway, mTOR signaling emerges as a central regulator of alveolar repair, metabolism, and fibrosis. As a master sensor of cellular nutrients and energy status, mTOR coordinates anabolic processes essential for epithelial regeneration [61]. Critically, mTOR not only functions in the cytoplasm but also translocates to the nucleus, where it acts as a transcriptional co‐activator to directly orchestrate gene expression programs governing cellular growth and metabolic reprogramming [62]. This nuclear function positions mTOR as a direct integrator of mechanical and metabolic cues in the alveolar microenvironment. Furthermore, hyperactivation of mTOR signaling is a recognized driver of pathological fibrosis across tissues, promoting the transition of fibroblasts to collagen‐secreting myofibroblasts and sustaining a profibrotic milieu [63].

In the context of BO, studies have identified significantly increased nuclear YAP protein and aberrant mTORC1‐ATF4 signaling transduction in BO lung tissue. mTORC1 signaling is essential for YAP/TAZ‐mediated upregulation of amino acid transporters (e.g., Slc7a5/Lat1), which promote AT2‐to‐AT1 differentiation. Notably, the YAP/TAZ‐mTORC1‐ATF4 axis is abnormally activated in the remodeled airway epithelium of BO, accompanied by a substantial increase in damage‐associated transient progenitors (DATPs) and AT1 cells, leading to severe pulmonary fibrosis [64]. This pathway offers a potential therapeutic target for BO and fibrotic lung diseases, potentially representing a novel approach for inhibiting pulmonary fibrosis.

## 5 Clinical Translation: From Mechanisms to Precision Intervention

Given the therapeutic resistance and late‐stage irreversibility of RLD pathology, the most effective treatment strategies must target the early pathological stages, or even implement preventive interventions before disease onset. Patients with advanced RLDs often present with irreversible pulmonary fibrosis accompanied by impaired AT2 function, which may lead to slow or stalled recovery post‐pneumonectomy. Therefore, treatment for late‐stage patients, besides mitigating fibrosis, often focuses on supportive care and enhancing cellular function. Furthermore, the heterogeneity among RLD patients underscores the need for more personalized therapeutic approaches. This section integrates current clinically promising treatment methods targeting the shared pathological mechanisms of RLDs.

### 5.1 Antioxidant Therapy

Targeting oxidative stress‐mediated damage (as detailed in Section 4.3.1 [6, 7]) is a promising strategy for RLDs. Although conventional antioxidants (e.g., vitamin E) show limited efficacy [65, 66], more potent agents like mitochondria‐targeted antioxidants offer potential, as discussed below.

N‐acetylcysteine (NAC), a tripeptide glutathione precursor, serves as a GSH (glutathione) reservoir, enhancing pulmonary antioxidant and antifibrotic capacity. Given that alveolar lavage fluid GSH levels are significantly reduced in mucus‐related diseases like IPF, early clinical trials suggested that NAC might alleviate oxidative damage and fibrosis progression in IPF patients, indicating its potential as an antioxidant and antifibrotic agent. However, subsequent larger trials such as the PANTHER‐IPF study revealed no significant clinical benefit from NAC monotherapy in broad IPF populations [67]. This conflicting evidence underscores a critical limitation in the field: the high heterogeneity of RLD patients. The therapeutic efficacy of antioxidants appears highly context‐dependent, with benefit confined to specific patient subsets (e.g., those carrying the TOLLIP rs3750920 TT genotype) [68]. This suggests that NAC's advantage lies in targeted GSH supplementation in GSH‐deficient cells, although it may be ineffective in GSH‐replete cells, highlighting the pitfalls of a one‐size‐fits‐all therapeutic approach. Nevertheless, studies on various CRDs have demonstrated that NAC can reduce oxidative stress and inflammation, leading to improved lung function in some cases [69]. Therefore, althoughg NAC remains a viable antioxidant therapy, its application must be guided by biomarker‐based patient stratification. The critical question of whether its efficacy can be enhanced through rational combination therapies to overcome resistance mechanisms requires further investigation.

Unlike the broad effects of NAC, targeted therapies exemplify precision medicine. Mitochondria‐targeted therapies exploit mitochondrial inner membrane potentials, using conjugation to lipophilic cations like triphenylphosphonium (TPP) to deliver compounds specifically to mitochondria [70]. MitoQ is a TPP‐based mitochondria‐targeted antioxidant. Compared to conventional nontargeted antioxidants (e.g., CoQ10), MitoQ specifically targets mitochondria and exhibits superior membrane permeability, offering antioxidant efficacy hundreds of times greater. It is currently being explored in cardiovascular and neurological disorders [71].

MitoQ holds substantial therapeutic promise for oxidative stress‐related lung diseases. Research by Cen et al. demonstrated that the mitochondria‐targeted antioxidant MitoQ, through specific modulation of the Nrf2‐MafF/antioxidant response element (ARE) signaling axis, significantly inhibits excessive production of mitochondrial ROS (mtROS). This effectively maintains mitochondrial membrane potential and ATP biosynthesis homeostasis and markedly reduces mitochondrial pathway‐mediated endothelial programmed cell death [47]. Simultaneously, MitoQ promotes Nrf2 nuclear translocation and enhances the transcriptional activity of the Nrf2/Keap1/ARE signaling pathway, preserving vascular endothelial permeability homeostasis. Ultimately, these actions confer multi‐faceted protection against acute lung injury by improving the structural integrity of the alveolar‐capillary membrane. A summary of these and other antioxidant therapies discussed in this section, including their mechanisms, current stages of research, and key findings, is provided in Table 1.

**TABLE 1:** Selected antioxidant therapies under investigation for refractory lung diseases.

Agent/Compound	Category	Mechanism of action	Stage of development (model)	Key findings/Clinical notes	Ref.
N‐acetylcysteine (NAC)	Glutathione precursor	Serve as a precursor for glutathione synthesis, enhancing cellular antioxidant capacity.	Clinical trials (IPF, COPD)	Mixed outcomes in major trials (e.g., PANTHER‐IPF). Efficacy may be confined to genetic subpopulations (e.g., TOLLIP rs3750920 TT genotype).	[67, 68, 69]
MitoQ (mitoquinone mesylate)	Mitochondria‐targeted antioxidant	TPP^+^ ‐conjugated ubiquinone that accumulates within mitochondria, scavenging mtROS and potentiating Nrf2 pathway activation.	Pre‐clinical (animal models of ALI/ARDS)	Demonstrate protection of alveolar‐capillary barrier integrity. Reduce inflammation and maintain mitochondrial bioenergetics.	[46, 47]
Vitamin E/Coenzyme Q10	Classical antioxidants	Scavenge free radicals and protect cellular membranes from lipid peroxidation.	Pre‐clinical/Limited clinical use	Demonstrate limited efficacy as a primary therapy for RLDs in clinical studies. Often utilized as a dietary supplement.	[65, 66]

*Note:* This table summarizes key agents discussed in the text and is not an exhaustive list.

Abbreviations: ALI/ARDS, acute lung injury/acute respiratory distress syndrome; COPD, chronic obstructive pulmonary disease; IPF, idiopathic pulmonary fibrosis; mtROS, mitochondrial reactive oxygen species.

### 5.2 Antifibrotic Therapy

Pulmonary fibrosis is driven by an aberrant repair cascade initiated by alveolar epithelial injury, particularly AT2 cell dysfunction [72]. This injury triggers profibrotic factor secretion (e.g., CCL2, TGF‐β) and fibroblast activation, leading to irreversible scar formation [52, 72, 73]. Concurrent AT2 senescence amplifies fibrosis via SASP [72]. Due to the irreversibility of pulmonary fibrosis, treatment options for advanced RLD patients are extremely limited, often restricted to lung transplantation, with quality of life post‐transplant being suboptimal.

Current mainstream antifibrotic drugs, pirfenidone (a TGF‐β inhibitor) and nintedanib (a multi‐target kinase inhibitor), suppress TGF‐β1‐induced fibroblast proliferation and ECM deposition [74]. They effectively slow the decline in lung function and show superior efficacy in IPF compared to commonly used corticosteroids or cyclosporine in some regions. However, they are unable to reverse established fibrosis or promote alveolar regeneration. Therefore, exploring novel strategies targeting early repair mechanisms holds significant clinical importance.

As previously mentioned, Wnt/β‐catenin signaling is a key driver of alveolar regeneration, but its systemic activation may promote fibroblast activation [75]. Recent experimental progress utilizing the CasRx RNA editing system has enabled controlled activation of lung epithelium‐specific Wnt signaling. This approach employs an AAV6 vector to deliver CasRx‐gRNA to lung epithelial cells, specifically degrading the mRNA of Axin1/Axin2‐scaffold proteins within the β‐catenin degradation complex, and resulting in transient Wnt pathway activation. This strategy increased AT2 cell proliferation (2‐fold increase in colony formation rate in organoids) and differentiation capacity toward AT1 cells (40% increase in AT1 coverage area in injury models), while reducing collagen deposition (35% decrease in hydroxyproline content) and myofibroblast activation levels (50% decrease in α‐SMA + cells), effectively promoting regeneration and exerting antifibrotic effects. The AAV6‐mediated effect subsided within 2 months, and lung epithelial Wnt activation did not cause significant side effects within 12 months [76]. This offers promising theoretical support for the clinical development of safe and potent early stage targeted antifibrotic drugs.

The specific thyroid hormone receptor β (TRβ) agonist sobetirome (GC‐1) also represents a potential AT2‐targeted therapeutic. The TRβ is a key regulator of AT2 cell differentiation. Experimental evidence demonstrates that GC‐1, in a bleomycin model, directly regulates KLF2 and CEBPA transcriptionally, activating the AT1 differentiation program (3‐fold increase in expression of AT1 markers Ager/Hopx). It simultaneously suppresses maladaptive AT2 (M‐AT2) cells, reducing KRT8+/CLDN4+ cell hyperplasia and paracrine fibroblast activation (60% decrease in collagen gel contraction). Notably, compared to pirfenidone, GC‐1 significantly improved survival rates without inducing hepatorenal toxicity [77].

AT2 functional impairment is a common pathological feature in RLDs. Research has identified that LRRK2 deficiency leads to impaired autophagy and accelerated senescence in AT2 cells. This recruits profibrotic macrophages (2‐fold increase in Arg1+ cells) via the CCL2/CCR2 axis, promoting fibrosis [72]. Thus, LRRK2 represents a potential intervention target. Restoring LRRK2 expression could potentially disrupt the AT2‐macrophage‐fibroblast pathogenic axis.

Although studies suggest AT1 cells possess potential for dedifferentiation into AT2 cells [12], for patients with advanced fibrosis, late‐stage irreversible lung injury is dominated by scar tissue. The remodeling capacity of senescent AT1 cells dedifferentiating into AT2 cannot affect the scar tissue itself. Therefore, the primary therapeutic goal for advanced pulmonary fibrosis patients is “fibrolysis”. This “fibrolysis‐regeneration” concept holds promise as a novel therapeutic approach for late‐stage RLD patients. Potential strategies include utilizing matrix metalloproteinase (MMP) activators or cysteine proteases to degrade mature collagen [78], creating space for regeneration. This could be combined with TRβ agonists (GC‐1) or CasRx‐Wnt editing to activate residual AT2 cell regeneration, aiming for lung tissue reconstitution. The characteristics of these discussed antifibrotic strategies, including their mechanisms, current stages of development, and key findings, are summarized in Table 2 for a comparative overview.

**TABLE 2:** Summary of current and investigational antifibrotic strategies for refractory lung diseases.

Strategy/Agent	Category	Mechanism of action	Stage of development (model)	Key findings/Notes	Ref.
Pirfenidone	Broad‐spectrum antifibrotic	Inhibit TGF‐β1‐induced fibroblast proliferation and ECM deposition.	Clinical use (IPF)	An approved standard‐of‐care for IPF. Effectively slow the decline in lung function. Cannot reverse established fibrosis.	[74]
Nintedanib	Multi‐target kinase inhibitor	Suppress signaling pathways involved in fibroblast activation (e.g., PDGF, FGF, VEGF receptors).	Clinical use (IPF, other progressive fibrotic ILDs)	An approved standard‐of‐care for IPF. Show superior efficacy compared to corticosteroids in some regions. Slow progression but do not reverse fibrosis.	[74]
GC‐1 (sobetirome)	Thyroid hormone receptor β (TRβ) agonist (pro‐regenerative)	Activate TRβ, promoting AT2‐to‐AT1 differentiation via transcriptional regulation of KLF2 and CEBPA.	Pre‐clinical (bleomycin‐induced lung fibrosis model)	Promote alveolar epithelial regeneration. Reduce KRT8+/CLDN4+ cell hyperplasia and fibroblast activation. Improve survival without hepatorenal toxicity in models.	[77]
AAV6‐CasRx system	Gene therapy (pro‐regenerative/antifibrotic)	AAV6 vector delivers CasRx‐gRNA to lung epithelial cells, specifically degrading Axin1/2 mRNA, resulting in transient Wnt/β‐catenin pathway activation.	Pre‐clinical (mouse model of lung injury)	Promote AT2 proliferation and differentiation while suppressing fibrosis. The effect is transient (About 2 months).	[76]
LRRK2 expression restoration	Targeted intervention (anti‐senescence)	Modulating LRRK2 expression to improve autophagy and anti‐senescence capacity in AT2 cells.	Pre‐clinical (research identification)	Represent a potential intervention target to disrupt the AT2‐macrophage‐fibroblast pathogenic axis.	[72]

Abbreviations: AT2, alveolar type II; FGF, fibroblast growth factor; ILDs, interstitial lung diseases; IPF, idiopathic pulmonary fibrosis; PDGF, platelet‐deriverd growth factor; TGF‐β, transforming growth factor‐beta; VEGF, vascular endothelial growth factor.

## 6 Integrating Mechanisms, Overcoming Barriers, and Advancing Toward Precision Medicine

### 6.1 Synthesis of Pathological Networks and Current Challenges

**FIGURE 3:**
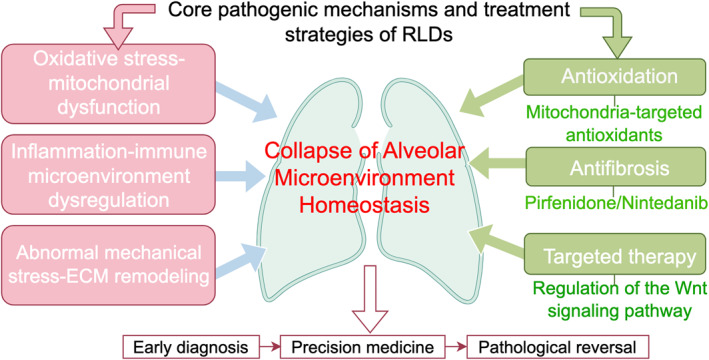
Overview of potential pathogenic conditions that lead to refractory lung diseases (RLDs). The core pathogenic mechanisms and therapeutic strategies of RLDs center on the “collapse of alveolar microenvironment homeostasis”. Oxidative stress—mitochondrial dysfunction, inflammation—immune microenvironment dysregulation, and abnormal mechanical stress‐extracellular matrix (ECM) remodeling, serving as core pathogenic mechanisms, drive the collapse of alveolar microenvironment homeostasis and facilitate the progression of RLDs. Antioxidation (such as mitochondria‐targeted antioxidants), antifibrosis (with pirfenidone and nintedanib being currently clinically available), and targeted therapy (e.g., regulation of the Wnt signaling pathway) target the pathogenic mechanisms to modulate and improve alveolar microenvironment homeostasis. The “counteraction” between these mechanisms and therapeutic strategies provides a translational framework for clinical practice, spanning from early diagnosis to precision medicine and culminating in the pathological reversal of RLDs.

As shown in Figure 3, the essence of RLDs lies in the collapse of alveolar microenvironmental homeostasis. Their pathological progression manifests as a bidirectional vicious cycle between initial alveolar structural injury and subsequent disease amplification. A deep understanding of the intricate architecture of the alveolus—particularly the reliance of the BGB on the mechanical support of type IV collagen and the tension modulation by surfactant to maintain its delicate equilibrium—is fundamental to overcoming therapeutic resistance in RLDs. However, it is crucial to critically appraise the limitations inherent in current research. Firstly, the majority of mechanistic insights are derived from preclinical models, which may not fully recapitulate the spatial‐temporal dynamics and cellular heterogeneity of human RLDs. For instance, the dormant macrophage phenotype in fibrotic niches was identified in a murine model, but its relevance to human disease progression requires further validation [3]. Secondly, the compelling paradigm of core pathological networks, while integrative, may oversimplify the extreme patient heterogeneity. Conflicting evidence regarding treatment efficacy, such as with NAC, underscores that our current molecular subtyping is still insufficient to reliably predict individual therapeutic responses [67, 68]. Lastly, for novel strategies like AAV‐mediated gene editing or “fibrolysis‐regeneration,” the challenges of delivery efficiency, off‐target effects, and long‐term safety present significant translational hurdles [76].

This review systematically synthesizes pathological evidence revealing that despite diverse clinical phenotypes across different RLDs, their underlying mechanisms converge on three core interwoven pathological networks: (1) oxidative stress coupled with mitochondrial dysfunction, with inhibition of the Nrf2/Keap1/ARE pathway acting as a key hub [7, 39, 40, 41, 44, 45]; (2) dysregulation of the inflammatory‐immune microenvironment, characterized by the cascade amplification of pro‐inflammatory cytokines (TNF‐α, IL‐1β, IL‐6, TGF‐β) and the vicious cycle driving fibrogenesis [8, 48, 49, 50, 51, 52]; and (3) aberrant mechanical stress signaling and matrix mechanotransduction remodeling, mediated by dysregulation of the Hippo‐YAP/TAZ pathway which senses ECM changes and dictates cell fate [53, 54, 55, 64]. These pathways and their crosstalk are detailed in Section 4.3. It is precisely this complex, network‐like regulatory mode that explains the limited efficacy of single‐target therapies (e.g., classical TGF‐β inhibitors) in patients with advanced disease, highlighting the urgent need for integrated interventions from a systems perspective and the implementation of stage‐specific, temporally sequenced therapeutic strategies.

### 6.2 Conclusion and Future Directions

Building upon this mechanistic integration, promising avenues for clinical translation are emerging, yet significant barriers remain. The foremost bottleneck is the lag in the therapeutic window. RLDs are often diagnosed only after the pathological process has progressed to an irreversible phase dominated by increased collagen deposition, rendering interventions aimed at “reversal” largely ineffective. Therefore, the core strategy for breaking this impasse lies in shifting the therapeutic window earlier. This necessitates the development of high‐sensitivity and specific diagnostic technologies targeting early, specific alveolar lesions, such as markers of AT2 cell dysfunction, BM injury, or activation of specific pathways. Leveraging single‐cell sequencing, spatial transcriptomics, imaging genomics, and artificial intelligence (AI) for risk stratification and early warning (e.g., identifying high‐risk individuals such as those with the TOLLIP rs3750920 TT genotype [67, 68]) is pivotal for enabling “preventative intervention before disease onset” or “intervention to prevent progression once disease is established”. This creates the opportunity for early and precise interventions (e.g., prophylactic use of the mitochondria‐targeted antioxidant MitoQ [46, 47]).

Regarding the development of innovative therapies, targeting the aforementioned core pathways shows immense potential. For diseases predominantly driven by oxidative damage (e.g., ARDS, BPD), the mitochondria‐targeted antioxidant MitoQ, which efficiently scavenges mtROS and potently activating the Nrf2 pathway, has been demonstrated in models to provide multidimensional protection to the BGB, holding substantial values for clinical translation [46, 47]. The promise for breaking through the bottleneck of antifibrotic therapy increasingly focuses on modulating the regenerative capacity of the alveolar epithelium, particularly AT2 cells. On one hand, strategies employing AAV6‐CasRx‐mediated, lung epithelium‐specific, transient degradation of Axin1/2 mRNA achieve controlled activation of the Wnt/β‐catenin pathway. This approach significantly promotes AT2 proliferation and differentiation while effectively suppressing fibrosis in models, providing a crucial proof‐of‐concept for developing safe and potent gene therapies [76]. On the other hand, the TRβ agonist GC‐1 promotes AT2‐to‐AT1 differentiation and suppresses pathological AT2 subsets and fibroblast activation through direct transcriptional regulation. Its efficacy surpassed that of pirfenidone in animal models, demonstrating superior safety, thus emerging as a highly promising oral small‐molecule pro‐regenerative drug [77]. Furthermore, restoring AT2 cell function (e.g., by modulating LRRK2 expression to improve autophagy and anti‐senescence capacity, thereby disrupting the AT2‐macrophage‐fibroblast profibrotic axis [72]) represents another vital intervention direction. For patients with established widespread irreversible fibrosis, exploring “fibrolysis‐regeneration” strategies embodies a paradigm‐shifting concept: utilizing matrix‐degrading enzymes (e.g., MMP activators [78]) to selectively degrade mature, cross‐linked collagen, thereby creating “regenerative niches” for residual alveolar stem cells (AT2). This can then be combined with potent pro‐regenerative therapies (e.g., localized CasRx‐Wnt editing [76] or GC‐1 [77]), theoretically enabling partial functional reconstruction of lung parenchyma. Furthermore, given the pivotal role of mTOR in integrating profibrotic signals, pharmacological inhibition of mTOR (e.g., with rapamycin analogs) represents a promising strategy to concurrently dampen epithelial dysfunction, metabolic dysregulation, and fibroblast activation [63]. However, the successful implementation of these strategies hinges critically on achieving precise spatial and temporal control, especially given the dual role of mTOR in both repair and fibrosis.

It must be emphasized that RLD patients exhibit significant clinical heterogeneity, mandating that future therapeutic approaches evolve towards personalized precision medicine. Molecular subtyping based on multi‐omics data (e.g., oxidative stress‐dominant, inflammation/fibrosis‐active, YAP/TAZ‐high, or mTOR‐hyperactive) will guide the precise selection of therapeutics, such as prioritizing MitoQ/Nrf2 activators, IL‐6/TGF‐β inhibitors or JAK inhibitors, Hippo pathway inhibitors, or mTOR inhibitors [63]. The mTOR signaling pathway, in particular, given its role as a nexus integrating metabolic, mechanical, and inflammatory cues, presents a compelling target for multi‐pathway intervention [61, 62, 63]. Concurrently, drug repositioning/repurposing strategies, such as a thorough exploration of expanding the indications of GC‐1 and other drugs for RLDs, represent an important avenue for rapidly accessing effective therapies [77].

Looking ahead, the definitive conquest of therapeutic resistance in RLDs depends on the deep integration of basic research, translational medicine, and clinical practice. Basic research must continue to dissect the dynamic network alterations within the alveolar microenvironment under pathological conditions (e.g., compounded by aging or infection) and the complex interactions between core pathways. Translational medicine should focus on developing more precise diagnostic tools, more efficient targeted delivery systems, and safer tissue regeneration strategies. At the clinical research level, there is a critical need to actively design basket or umbrella trials based on refined molecular subtyping for early stage patients. These trials should validate the synergistic effects of combination therapies and the superiority of personalized treatment regimens. Only through such multidisciplinary, multilayered, and integrated efforts can we overcome the current impasse and deliver truly effective precision treatments for patients suffering from RLDs. The ultimate goal is to shift from merely “controlling symptoms” to achieving “pathological reversal,” and from “prolonging survival” to “restoring function.”

## Author Contributions

L.Z. and J.F. conceived and designed the study. Y.Q., X.D., Y.Z., N.M., A.C., Y.P., S.Z., and M.X. participated in literature data collection and manuscript writing. Y.Q., L.Z., and J.F. drafted and revised the manuscript. All authors reviewed, edited, and approved the manuscript.

## Conflicts of Interest

The authors declare no conflicts of interest.

## Data Availability

Data sharing is not applicable to this article as no datasets were generated or analyzed during the current study.
